# Associations between continuous health examinations and trajectories of comorbidity of chronic disease: Evidence from a nationwide longitudinal study of middle-aged and older adults in China

**DOI:** 10.1016/j.ghrp.2026.06.006

**Published:** 2026-06-29

**Authors:** Yizhou Ao, Jing Wang, Dan Han, Sangsang Li, Shiping Gu, Xinnan Du, Yixuan Zhong

**Affiliations:** a Department of Health Management, School of Medicine and Health Management, Tongji Medical College, Huazhong University of Science and Technology, Wuhan, Hubei 430030, China; b The Key Research Institute of Humanities and Social Science of Hubei Province, Huazhong University of Science and Technology, Wuhan, Hubei 430030, China

**Keywords:** Health Examinations, Chronic disease comorbidity, Latent class growth analysis, China, CHARLS

## Abstract

**Background:**

As population aging accelerates, the escalating prevalence of comorbidity of chronic diseases (CCD) necessitates evidence-based preventive strategies. This study aims to examine associations between continuous participation in health examinations and the long-term CCD trajectories among Chinese adults aged 45 and older, providing evidence to optimize targeted prevention.

**Methods:**

This study used data from four waves of China Health and Retirement Longitudinal Study (CHARLS), specifically 2013, 2015, 2018, and 2020. Latent class growth analysis was used to classify trajectories of CCD. Chi-square tests and multivariate logistic regression were conducted to examine the relationship between CCD and health examination behaviors. A regression discontinuity design was also implemented to assess the impact of China’s free health examination policy.

**Results:**

A total of 12,510 participants data were categorized into six latent classes. In the unadjusted model, continuous health examination participants were significantly more likely to be classified into the Health Risk (Relative risk ratio (RRR) = 2.249; 95% CI: 1.780–2.842) and Multiple Chronic Diseases Worsening groups (RRR = 7.124; 95% CI: 5.454–9.305) compared to non-participants. After adjusting for baseline burden, individuals with a low baseline burden who participated in health examination had a lower likelihood of unfavorable CCD trajectories (RRR = 0.456; 95% CI: 0.278–0.748; *P* < 0.01) than non-participants. Older adults were more likely to participate in continuous examinations (OR = 21.571; 95% CI: 11.732–39.662). After controlling for sociodemographic factors, the association between continuous health examinations and CCD trajectories varied by baseline burden, showing stronger effects in individuals with lower baseline burden and weaker effects in those with higher burden (*P* < 0.01).

**Conclusions:**

Continuous health examinations are significantly associated with CCD trajectories. However, their effectiveness remains moderated by baseline disease burden. The current free health examination policy effectively fosters CCD management, yet this association is contingent upon age and socioeconomic determinants. The extension of eligibility to individuals below 65 could maximize the potential for early chronic disease control. This may also serve as a valuable blueprint for other low- and middle-income countries for optimizing cost-effectiveness of their preventive healthcare systems before populations age into high-burden morbidity.

## Introduction

Non-communicable diseases and their comorbidities present a profound challenge to global public health systems.^1^ Comorbidity of chronic diseases (CCD), defined as the coexistence of two or more chronic conditions,^2^ affects a substantial proportion of the aging population worldwide.^3^ The compounding effects of CCD substantially amplify the risk of adverse health outcomes, including premature mortality, frequent hospital admissions, and prolonged stays, while imposing a severe economic burden on healthcare systems by accounting for 78% of primary care consultations in high-income countries and driving an exponential surge in care costs.^4^ Therefore, CCD progression is a global health priority that requires in-depth exploration, particularly for low- and middle-income countries experiencing rapid demographic transitions. In China, population aging and unhealthy behaviors are prevalent,^5^ and the burden of chronic diseases in China has been steadily increasing.^6^ Currently, the prevalence of CCD among individuals aged 45 and older has reached 55.77%,^7^ indicating that it has become a significant issue for middle-aged and older adults and may substantially increase future risks for the public health system.^8^ Consequently, examining CCD trajectories in China offers valuable empirical evidence for optimizing global health strategies.

The progression of CCD is highly complex and profoundly influenced by an individual's baseline chronic disease burden.^9^, ^10^ Given this complexity, effective early intervention is crucial for mitigating disease progression and reducing overall public health risks.^11^ Regular health examinations serve as a globally recognized strategy to achieve this through the facilitation of early detection and promotion of sustained behavioral changes, ultimately lowering healthcare costs.^12^ While the economic benefits of general health examinations remain debated, early detection and prevention generally improve economic outcomes and reduce the overall burden of chronic diseases.^13^, ^14^ Furthermore, continuous health examinations offer greater benefits in improving health literacy, promoting behavioral changes, reducing healthcare costs, and supporting the proactive management of chronic diseases, especially CCD. The evidence from China confirms that continuous engagement in health examinations significantly reduces long-term healthcare expenditures, similar to global findings.^15^

To address the growing chronic disease burden, China launched the National Essential Public Health Services (NEPHS) program in 2009, offering free health examinations and chronic disease follow-up for individuals aged 65 and above. Subsequent national initiatives, such as the Healthy China 2030 blueprint, have further expanded these services to emphasize standardized health management and accessible preventive guidance, reinforcing long-term support for older adults.

While existing evidence indicates that free health examination policies help reduce healthcare costs and lower chronic disease risks,^14^, ^16^ few studies have investigated the long-term associations between continuous health examinations and CCD trajectories. Most prior research has focused only on short-term outcomes, the evidence that continuous health examinations can alter the longitudinal evolution of CCD requires further validation, and whether current free health examination policies adequately alleviate disease progression remains unclear.^17^ Furthermore, because traditional single-variable classifications fail to fully capture the dynamic complexity of chronic disease trajectories, identifying high-risk individuals requires advanced longitudinal approaches. To address this, this study utilizes Latent Class Growth Analysis (LCGA) to group individuals based on similar health trajectories, providing a clearer understanding of dynamic CCD development and its potential associations with continuous health examinations and to identify the longitudinal trajectory patterns and subgroup characteristics of CCD among middle-aged and older adults in China. By addressing this gap, the findings are expected to provide valuable empirical evidence for optimizing domestic health examination policies and for informing broader global health strategies and preventive interventions for chronic disease management in aging populations worldwide, particularly in other low- and middle-income countries.

## Methods

### Study design

This study employed a longitudinal observational design to investigate the association between continuous health examination behaviors and the trajectories of chronic continuous diseases (CCD). To comprehensively capture this dynamic process, we initially utilized LCGA to identify unobserved heterogeneity in chronic disease progression and establish distinct CCD trajectories over time. Building upon these classifications, the study examined the associations between health examination behaviors and the identified trajectories, with a specific focus on the moderating role of baseline disease burden. Because the effectiveness of continuous health examinations was found to be closely related to this baseline status, the analysis further explored the determinants of continuous health examination participation. Recognizing the critical role of age and China’s policy that provides free health examinations to individuals aged 65 and above, we incorporated a Regression Discontinuity Design (RDD) and sub-sample analyses to isolate the specific impact of the policy and evaluate the factors driving continuous preventive care engagement.

### Data source

The data from the China Health and Retirement Longitudinal Study (CHARLS) was used for the study. CHARLS is a nationally representative longitudinal survey targeting individuals aged 45 and older in China. As of November 2023, the study had conducted five waves of follow-up surveys across 150 counties and 450 communities or villages in 28 provinces (autonomous regions, and municipalities). The survey waves were conducted in 2011, 2013, 2015, 2018, and 2020, involving a total of 10,204 households and 17,364 respondents. However, this study utilized data from 2013 to 2020 to ensure longitudinal consistency, as the design of key variables in the first wave differed significantly from that in subsequent years.

**Fig. 1:**
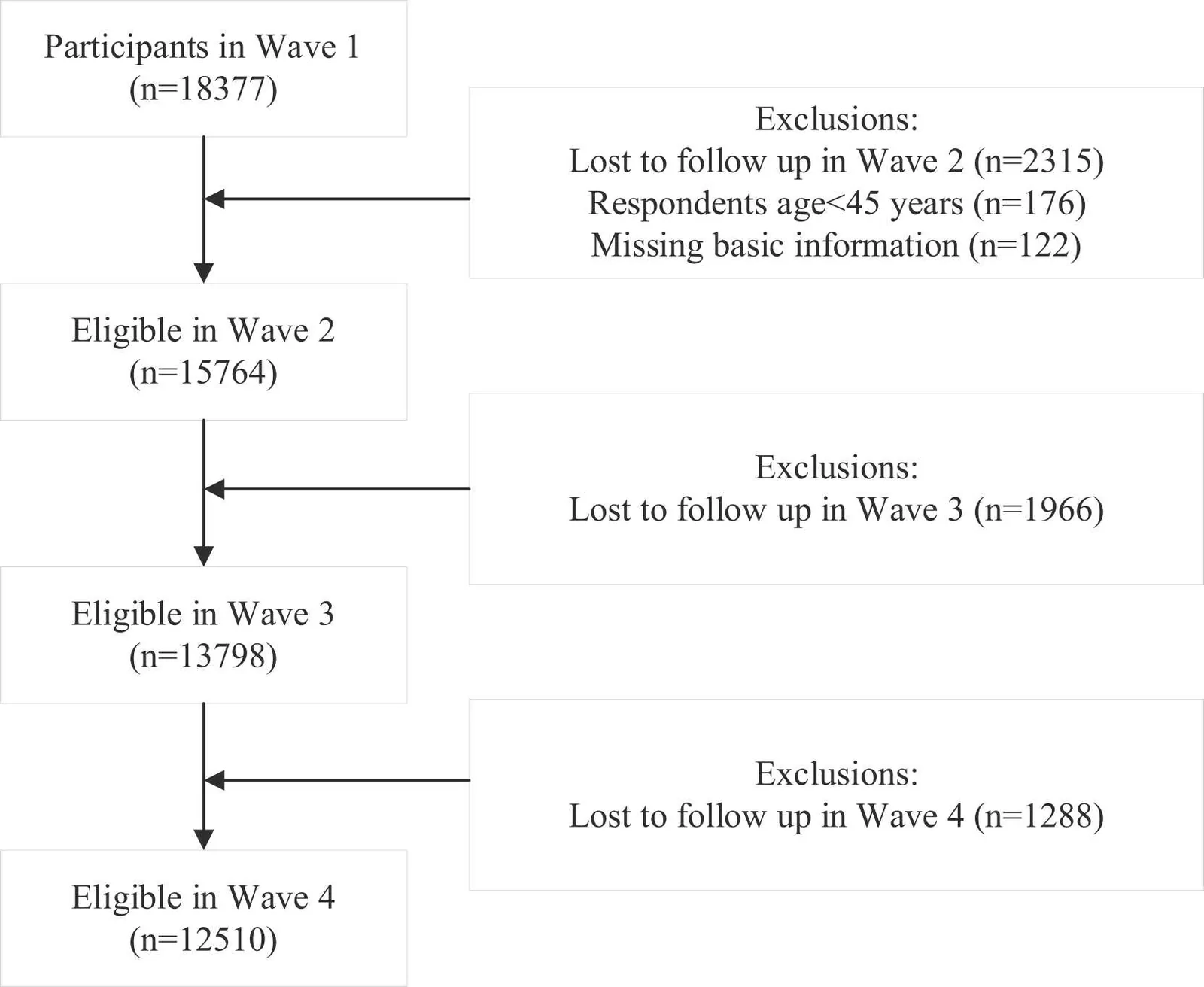
Flowchart of participant selection.

For this study, we selected individuals aged 45 years and above who participated in all four survey waves from 2013 to 2020. After excluding participants data with missing key variables (e.g., demographic characteristics, health examination records, or chronic disease diagnoses), the final analytical sample consisted of 12,510 individuals (5841 males and 6669 females). Fig. 1 shows the sample selection process.

### Variables

In this study, the dependent variable was the latent class of the change in the number of chronic diseases among respondents from 2013 to 2020. Chronic diseases were categorized into 14 types, including hypertension, dyslipidemia, diabetes or increased blood sugar levels, cancer or malignant tumor, chronic lung disease, liver disease, heart disease, stroke, kidney disease, stomach or other digestive disease, emotional or psychiatric problems, memory-related diseases, arthritis or rheumatism, and asthma, to determine the number of chronic diseases. LCGA was conducted to classify respondents into distinct latent classes and to analyze the number of chronic diseases based on their disease trajectory patterns. The number of chronic diseases was capped at nine to ensure model stability and align with methodological precedents in multimorbidity research.^18^, ^19^ This was done to reduce the influence of extreme outliers and prevent the data from being significantly right-skewed, as very few individuals in our sample reported more than nine comorbidities. Based on LCGA results, respondents were categorized into six distinct subgroups according to their CCD trajectory patterns. Based on the respondents' baseline chronic disease status, baseline chronic diseases were categorized into three groups: 0, 1–2, and 3 or more. Similarly, changes in CCD were classified into three levels: 0, 1–2, and 3 or more (Supplementary Table 2).

Health examination was selected as the primary independent variable. Participation was assessed in each survey wave using the CHARLS question: “Since your last interview, when did you receive the latest physical examination.” The questionnaire distinguishes preventive health examinations from diagnostic screenings during treatment. Although specific clinical items are not listed in the CHARLS, the most accessible health examination services for this study population are highly standardized and align with the NEPHS. These examinations typically include physical assessments, laboratory tests (blood and urine routines, fasting blood glucose, lipids, and liver/kidney function), and basic imaging assessments (electrocardiograms and abdominal ultrasound). Based on respondents’ participation across four survey waves (2013, 2015, 2018, and 2020), health examination behaviors were classified into three categories: never participated in health examinations, participated in health examinations but not continuously, and participated in health examinations continuously.

### Covariate assessments

The covariates included respondents' sociodemographic characteristics (sex, age, educational level, area of residence, marital status, and insurance status), family characteristics (number of children, children’s highest educational level, and co-residence with children), health status (activities of daily living, [ADL]), lifestyle behaviors (smoking and drinking habits), and insurance status (detailed coding information is provided in Supplementary Table 1).

### Statistical analysis

A chi-square test was used to examine the differences in CCD trajectories across various health examination behaviors within the entire sample. To construct these trajectories, LCGA was conducted to identify distinct longitudinal subgroup patterns. The optimal number of latent classes was determined iteratively based on clinical interpretability and model fit indices, including Akaike Information Criterion (AIC), Bayesian Information Criterion (BIC), sample-size adjusted BIC, Vuong-Lo-Mendell-Rubin likelihood ratio test (VLMR), Bootstrapped Likelihood Ratio Test (BLRT), and Entropy. Based on these identified trajectories, multivariate logistic regression was then employed to examine their associations with health examination behaviors while adjusting for individual-level covariates. A Regression Discontinuity Design (RDD) was implemented to further isolate the specific impact of the free health examination policy for adults aged 65 and older. An additional sub-sample analysis was conducted specifically focusing on individuals aged 65 and older, building upon the findings of the RDD analysis to control for potential confounding effects of age and policy exposure on CCD trajectories. Finally, a sensitivity analysis utilizing a quasi-weighted index based on the Charlson Comorbidity Index (CCI) was performed to verify the robustness of the primary findings. LCGA was executed in Mplus 8.1 (Muthen & Muthen, Los Angeles, U.S.), while other analyses were performed using SPSS 27.0 and Stata 17.0.

## Results

### CCD Trajectory Identification and Descriptive Statistics

A six-class model was selected as the optimal solution for CCD trajectories, based on the LCGA model fit indices and interpretability.

**Fig. 2:**
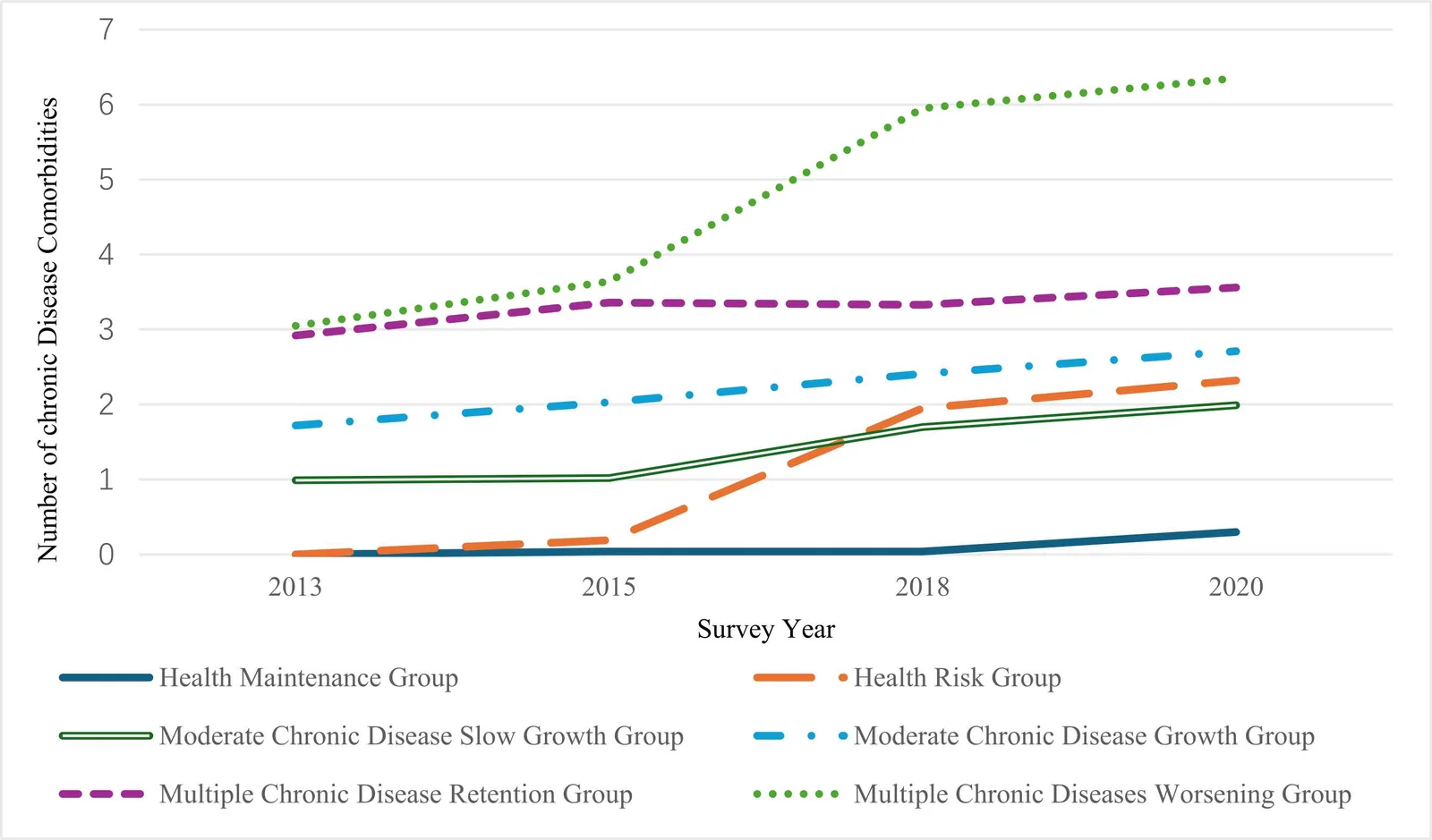
Distribution of Chronic Disease Trajectories Across Latent Groups.

Fig. 2 illustrates the distribution of chronic disease counts across six latent classes. The Health Maintenance group (13.65%) comprised individuals who did not have chronic diseases at baseline and a stable trajectory. In contrast, the Health Risk group (20.44%) included individuals who did not initially have chronic diseases but experienced rapid health deterioration. The Moderate Chronic Disease Slow Growth group (23.73%) comprised individuals with a small number of chronic diseases at baseline, showing a slow and stable trajectory. Meanwhile, the Moderate Chronic Disease Growth group (18.31%) had a higher baseline disease burden but followed a similar moderate health deterioration trajectory. The Multiple Chronic Disease Retention group (11.97%) had a high initial burden but remained relatively stable. Finally, the Multiple Chronic Diseases Worsening group (11.90%) showed both high baseline burden and a severely worsening trajectory.

### Associations between health examination behaviors and CCD trajectories

As shown in Table 1, Most participants lived in rural areas (81.1%). A balanced sex distribution was observed (46.7% male, 53.3% female), and 26.0% were aged 65 or more. The sample sizes for the six latent classes were as follows: 1708, 2557, 2968, 2290, 1498 and 1489. Continuous health examinations were associated with fewer chronic diseases (χ² = 184.805, *P* < 0.001). Men and rural residents experienced greater increases in chronic disease (χ² = 50.324, *P* < 0.001; χ² = 29.589, *P* < 0.001). Moreover, differences in CCD trajectories were statistically significant across different health insurance types (χ² = 60.429, *P* < 0.01) and were also associated with smoking and drinking behaviors.

**Table 1:** Descriptive statistics.

**Variable**	**Total**	**Chronic Disease Latent Class**						$\chi^{2}$**/F**	***p***
	(n = 12,510)	Health Maintenance Group (n = 2968)	Health Risk Group (n = 1708)	Moderate Chronic Disease Slow Growth Group (n = 1498)	Moderate Chronic Disease Growth Group (n = 1489)	Multiple Chronic Disease Retention Group (n = 2557)	Multiple Chronic Diseases Worsening Group (n = 2290)		
Chronic Disease Latent Class (%)		13.65	20.44	23.73	18.31	11.97	11.90		
Baseline chronic diseases (Mean)	59.18	0.00	0.00	0.99	1.72	2.92	3.05	6660.160	< 0.001
Sex (%)								55.894	< 0.001
Female	53.31	12.48	19.67	23.02	18.64	12.91	13.29		
Male	46.69	15.00	21.31	24.53	17.93	10.91	10.32		
Age (Mean)	59.17	56.51	58.25	58.79	60.00	61.15	61.33	73.750	< 0.001
Urbanity (%)								45.636	< 0.001
Rural	81.10	13.92	20.34	24.32	18.50	11.87	11.05		
Urban	18.90	12.52	20.85	21.15	17.47	12.44	15.57		
Education level (%)								107.304	< 0.001
Illiteracy	26.17	10.72	19.85	24.77	18.63	13.35	12.68		
Primary school	40.79	12.80	19.34	23.38	19.54	12.52	12.42		
Middle school and above	33.04	17.03	22.26	23.32	16.53	10.21	10.65		
Number of children (Mean)	2.62	2.38	2.52	2.59	2.69	2.79	2.80	25.010	< 0.001
Child Education (%)								21.7106	< 0.01
Primary or below	16.66	10.38	18.47	24.36	19.09	13.63	14.07		
Junior high School	36.45	12.98	19.25	22.31	19.98	12.74	12.74		
Senior high school and above	46.89	13.81	20.31	23.46	18.06	10.90	13.46		
Child Reside (%)								62.662	< 0.001
Not living with any child	46.03	12.33	19.03	23.26	18.70	13.04	13.64		
Living with at least one child	53.97	14.77	21.73	24.01	18.05	11.05	10.40		
Marital status (%)								29.820	< 0.001
Unmarried	10.70	11.14	18.54	22.05	19.13	14.20	14.95		
Married	89.30	13.95	20.67	23.93	18.21	11.71	11.54		
Insurance (%)								79.798	< 0.001
Urban employee medical insurance	11.31	11.45	21.63	21.20	17.10	12.79	15.83		
Urban and rural resident medical insurance	84.95	13.62	20.08	24.11	18.74	12.00	11.46		
Others	0.55	24.64	21.74	18.84	5.80	14.49	14.49		
No insurance	3.19	20.55	25.56	23.31	13.28	8.02	9.27		
ADL								623.107	< 0.001
No difficulty	84.90	15.50	21.54	24.57	17.95	10.75	9.68		
Difficulty in at least one ADL item	15.10	3.28	14.24	18.95	20.28	18.85	24.40		
Drinking behavior (%)								165.439	< 0.001
No drinking	49.22	12.04	19.59	22.72	19.07	12.98	13.61		
Non-continuous drinking	31.44	12.97	20.57	24.33	18.10	11.54	12.48		
Continuous drinking	19.34	18.88	22.40	25.29	16.69	10.12	6.61		
Smoking behavior (%)								78.189	< 0.001
No smoking	64.35	12.56	19.70	23.54	18.96	12.65	12.60		
Non-continuous smoking	24.90	14.29	21.28	23.92	17.37	11.40	11.75		
Continuous smoking	10.75	18.74	22.90	24.39	16.58	9.29	8.10		
Examination (%)								321.979	< 0.001
Never participated in health examinations	20.61	21.76	20.25	25.80	16.45	9.46	6.28		
Participated in health examinations but not continuously	66.61	12.08	20.90	23.12	18.86	12.55	12.47		
Participated in health examinations continuously	12.78	8.76	18.32	23.51	18.39	13.01	18.01		

Table 2 presents the results of multiple logistic regression analyses examining the associations between health examination behavior and CCD classifications. In the unadjusted model (Table 1), individuals with non-continuous examinations showed a higher likelihood of being classified into the Health Risk (RRR=1.859; 95% CI: 1.613–2.143) and Multiple Chronic Diseases Worsening groups (RRR=3.573; 95% CI: 2.94–4.343) than that of non-participants. Continuous examinations were associated with even higher likelihoods (Health Risk: RRR=2.249; 95% CI: 1.78–2.842; Multiple Chronic Diseases Worsening: RRR=7.124; 95% CI: 5.454–9.305), and after adjusting for key sociodemographic covariates, associations remained significant. The stronger association was observed among those with continuous examinations (Multiple Chronic Diseases Worsening: RRR = 5.189; 95% CI: 3.884–6.932).

**Table 2:** Associations between Health Examination Continuity and CCD Trajectory Groups (2013–2020).

**Variables**	**Options**	**Chronic Disease Latent Class**									
		**Model 1 (Reference: Health Maintenance Group)**					**Model 2 (Reference: Health Maintenance Group)**				
		**RRR (95%CI)**					**RRR (95%CI)**				
		Health Risk Group	Moderate Chronic Disease Slow Growth Group	Moderate Chronic Disease Growth Group	Multiple Chronic Disease Retention Group	Multiple Chronic Diseases Worsening Group	Health Risk Group	Moderate Chronic Disease Slow Growth Group	Moderate Chronic Disease Growth Group	Multiple Chronic Disease Retention Group	Multiple Chronic Diseases Worsening Group
Examination (Never participated in health examinations)	Participated in health examinations but not continuously	1.859*** (1.613,2.143)	1.614*** (1.409,1.849)	2.065*** (1.78,2.397)	2.388*** (2.008,2.841)	3.573*** (2.94,4.343)	1.739*** (1.501,2.015)	1.483*** (1.287,1.709)	1.807*** (1.545,2.112)	1.946*** (1.622,2.334)	2.909*** (2.368,3.574)
	Participated in health examinations continuously	2.249*** (1.78,2.842)	2.266*** (1.811,2.835)	2.779*** (2.191,3.523)	3.416*** (2.629,9.305)	7.124*** (5.454,9.315)	2.003*** (1.561,2.570)	2.041*** (1.606,2.593)	2.282*** (1.769,2.944)	2.537*** (1.914,3.362)	5.189*** (3.884,6.932)
Sex (Male)	Female	-	-	-	-	-	1.103 (0.916,1.328)	1.171 (0.978,1.404)	1.105 (0.914,1.337)	0.985 (0.797,1.216)	0.924 (0.747,1.145)
Age		-	-	-	-	-	1.010** (1.000,1.020)	1.014*** (1.004,1.023)	1.028*** (1.017,1.038)	1.034*** (1.023,1.046)	1.024*** (1.012,1.035)
Urbanity (Rural)	Urban	-	-	-	-	-	1.052 (0.848,1.304)	1.002 (0.810,1.239)	1.149 (0.919,1.437)	1.158 (0.902,1.487)	1.525*** (1.193,1.948)
Education level (No formal education)	Primary school	-	-	-	-	-	0.885 (0.741,1.056)	0.873 (0.735,1.037)	1.092 (0.911,1.308)	1.092 (0.897,1.330)	1.114 (0.911,1.363)
	Middle school and above	-	-	-	-	-	0.778** (0.637,0.950)	0.707*** (0.582,0.859)	0.817 (0.664,1.006)	0.812 (0.644,1.025)	0.831 (0.656,1.053)
Number of children		-	-	-	-	-	1.027 (0.967,1.091)	1.051 (0.991,1.115)	1.049 (0.987,1.116)	1.078** (1.009,1.152)	1.109*** (1.037,1.187)
Child Education (Primary or below)	Junior high School						0.895 (0.681,1.175)	0.792 (0.609,1.029)	0.911 (0.693,1.197)	0.844 (0.627,1.135)	0.801 (0.594,1.081)
	Senior high school and above						0.890 (0.679,1.167)	0.825 (0.636,1.070)	0.812 (0.617,1.067)	0.714** (0.529,0.963)	0.767 (0.569,1.034)
Child Residence (Not living with any child)	Living with at least one child						1.039 (0.912,1.185)	0.961 (0.846,1.092)	0.951 (0.831,1.088)	0.870 (0.749,1.010)	0.783*** (0.672,0.912)
Marital status (Unmarried)	Married	-	-	-	-	-	1.070 (0.838,1.366)	1.158 (0.912,1.471)	1.263 (0.984,1.620)	1.144 (0.875,1.497)	1.239 (0.946,1.622)
Insurance (Urban employee medical insurance)	Urban and rural resident medical insurance	-	-	-	-	-	0.780 (0.593,1.024)	0.906 (0.690,1.190)	0.933 (0.701,1.242)	0.754 (0.551,1.032)	0.746 (0.549,1.014)
	Others	-	-	-	-	-	0.437** (0.207,0.923)	0.408** (0.190,0.877)	0.160*** (0.052,0.493)	0.507 (0.217,1.185)	0.362** (0.145,0.907)
	No insurance	-	-	-	-	-	0.697 (0.472,1.029)	0.610** (0.411,0.907)	0.429*** (0.273,0.675)	0.338*** (0.200,0.569)	0.426*** (0.258,0.704)
ADL (No difficulty)	Difficulty in at least one ADL item						2.914*** (2.174,3.904)	3.343*** (2.512,4.447)	4.585*** (3.444,6.104)	6.924*** (5.177,9.259)	10.194*** (7.642,13.598)
Drinking behavior (No drinking)	Non-continuous drinking	-	-	-	-	-	0.959 (0.821,1.120)	1.010 (0.868,1.175)	0.926 (0.790,1.086)	0.886 (0.743,1.057)	0.896 (0.752,1.069)
	Continuous drinking	-	-	-	-	-	0.755*** (0.626,0.911)	0.778*** (0.648,0.935)	0.653*** (0.536,0.796)	0.639*** (0.512,0.799)	0.387*** (0.304,0.494)
Smoking behavior (No smoking)	Non-continuous smoking	-	-	-	-	-	1.024 (0.852,1.231)	0.929 (0.777,1.111)	0.878 (0.726,1.062)	0.980 (0.794,1.210)	1.163 (0.939,1.441)
	Continuous smoking	-	-	-	-	-	0.922 (0.739,1.150)	0.762** (0.613,0.948)	0.744** (0.588,0.942)	0.713** (0.542,0.937)	0.809 (0.609,1.076)

*P < 0.05, **P < 0.01, and ***P < 0.001. RRR: Relative Risk Ratio; 95% CI= 95% Confidence Interval.

Among the control variables, older age (*P* < 0.001), continuous smoking (*P* < 0.01), urban residence (RRR=1.525), and increased number of children (RRR = 1.109; 95% CI: 1.037–1.187) were associated with higher risks of unfavorable trajectories. Conversely, higher education (RRR=0.707; 95% CI: 0.582–0.859), continuous alcohol consumption (RRR=0.387; 95% CI: 0.303–0.494), and living with children (RRR = 0.783; 95% CI: 0.672–0.912) were associated with a lower likelihood of classification into high-risk groups. Notably, ADL exhibited a strong association with a worsening trajectory (RRR = 10.194; 95% CI: 7.642–13.598).

### Stratified analysis by baseline chronic disease burden

Models 3, 4, and 5 in Table 3 analyze the associations between health examination behavior and CCD trajectories in individuals with different baseline disease burden. Among those with a low baseline burden (Model 3), both non-continuous (RRR = 1.739, 95% CI: 1.501–2.015) and continuous participants (RRR = 2.003, 95% CI: 1.561–2.570) were significantly more likely to be classified into the Health Risk group than non-participants.

**Table 3:** Associations between Continuous Health Examinations and CCD Trajectories across Different Baseline Chronic Diseases Levels.

Variables	Options	Chronic Disease Latent Class		
		Model 3	Model 4	Model 5
		RRR (95% CI)		
		Health Risk Group (Reference: Health Maintenance Group)	Moderate Chronic Disease Growth Group (Reference: Moderate Chronic Disease Slow Growth Group)	Multiple Chronic Diseases Worsening Group (Reference: Multiple Chronic Disease Retention Group)
Examination (Never participated in health examinations)	Participated in health examinations but not continuously	1.739*** (1.501,2.015)	1.218** (1.055,1.406)	1.495*** (1.197,1.868)
	Participated in health examinations continuously	2.003*** (1.561,2.570)	1.118 (0.908,1.377)	2.046*** (1.542,2.714)
Sex (Male)	Female	1.103 (0.915,1.328)	0.944 (0.805,1.106)	0.939 (0.763,1.156)
Age		1.010 (1.000,1.020)	1.014*** (1.006,1.022)	0.990 (0.979,1.001)
Urbanity (Rural)	Urban	1.052 (0.848,1.304)	1.147 (0.943,1.394)	1.316* (1.029,1.684)
Education level (No formal education)	Primary school	0.885 (0.741,1.056)	1.250** (1.080,1.447)	1.020 (0.844,1.232)
	Middle school and above	0.778* (0.637,0.950)	1.155 (0.968,1.379)	1.023 (0.807,1.297)
Number of children		1.027 (0.967,1.091)	0.998 (0.950,1.447)	1.029 (0.967,1.095)
Child Education (Primary or below)	Junior high School	0.895 (0.681,1.175)	1.150 (0.925,1.431)	0.950 (0.722,1.250)
	Senior high school and above	0.889 (0.679,1.167)	0.984 (0.790,1.225)	1.074 (0.813,1.419)
Child Residence (Not living with any child)	Living with at least one child	1.039 (0.912,1.185)	0.989 (0.882,1.109)	0.900 (0.774,1.047)
Marital status (Unmarried)	Married	1.069 (0.838,1.366)	1.090 (0.888,1.339)	1.083 (0.844,1.389)
Insurance (Urban employee medical insurance)	Urban and rural resident medical insurance	0.779 (0.593,1.024)	1.029 (0.806,1.316)	0.989 (0.732,1.337)
	Others	0.437* (0.207,0.923)	0.391 (0.125,1.222)	0.715 (0.273,1.871)
	No insurance	0.697 (0.472,1.029)	0.704 (0.461,1.074)	1.262 (0.717,2.221)
ADL (No difficulty)	Difficulty in at least one ADL item	2.913*** (2.174,3.904)	1.372*** (1.168,1.610)	1.472*** (1.244,1.743)
Drinking behavior (No drinking)	Non-continuous drinking	0.958 (0.821,1.120)	0.917 (0.805,1.110)	1.012 (0.852,1.201)
	Continuous drinking	0.755*** (0.626,0.911)	0.839* (0.706,0.998)	0.606*** (0.471,0.779)
Smoking behavior (No smoking)	Non-continuous smoking	1.024 (0.852,1.231)	0.945 (0.805,1.110)	1.187 (0.962,1.466)
	Continuous smoking	0.922 (0.739,1.150)	0.976 (0.790,1.206)	1.135 (0.838,1.537)

*P < 0.05, **P < 0.01, and ***P < 0.001, 95% CI= 95% confidence interval for the odds ratio.

The associations with health examinations varied based on baseline chronic disease status for other CCD groups. In the moderate baseline group (Model 4), those who received non-continuous health examinations were more likely to be categorized into the Moderate Chronic Disease Growth group (RRR = 1.218, 95% CI: 1.055–1.406). However, continuous health examinations did not show a significant association (RRR = 1.118, 95% CI: 0.908–1.377). In the high baseline burden group (Model 5), both non-continuous (RRR = 1.495, 95% CI: 1.197–1.868) and continuous participation (RRR = 2.046, 95% CI: 1.542–2.714) increased the risk of a worsening trajectory.

Age and ADL limitation were consistently associated with unfavorable trajectories (*P* < 0.001). Regarding socioeconomic factors, urban residence was associated with a higher risk of worsening trajectories in the high-burden group (RRR = 1.316, 95% CI: 1.029–1.684). Patients with higher education (RRR = 0.778, 95% CI: 0.637–0.950) were significantly less likely to be categorized into the Health Risk group.

### Policy impact and determinants of continuous health examination participation

As shown in Table 4, Model 6 presents the results of an RDD that evaluated the overall association between age and the 65-year policy cutoff. Individuals aged 65 and above showed a higher likelihood of participating in continuous health examinations (OR = 21.571, 95% CI: 11.732–39.662, *P* < 0.001), and this likelihood was positively associated with each additional year of age (OR = 1.047, 95% CI: 1.041–1.053, *P* < 0.001). However, the interaction term suggests that among individuals aged 65 and older, the positive association between age and participation in continuous health examinations weakens as age increases (OR = 0.961, 95% CI: 0.952–0.969, *P* < 0.001).

**Table 4:** Regression Discontinuity Analysis of Associations between Age and Free Health Examination Policy with Continuous Participation among Older Individuals.

Variables	Options	Model 6	Model 7
		OR (95% CI)	OR (95% CI)
		Participated in health examinations continuously (Reference: Participated in health examinations but not continuously)	Participated in health examinations continuously (Reference: Participated in health examinations but not continuously)
Breakpoint Variable (Age)	> 65 years	21.571*** (11.732,39.662)	4.223 (0.156,114.389)
	Age in Years	1.047*** (1.041,1.053)	1.107*** (1.067,1.148)
	Interaction (>65 × Age)	0.961*** （0.952,0.969）	0.978 (0.929,1.029)
Sex (Male)	Female	0.964 (0.898,1.028)	0.915 (0.816,1.026)
Urbanity (Rural)	Urban	1.241*** (1.145,1.346)	1.400*** (1.211,1.619)
Education level (No formal education)	Primary school	1.131*** (1.064,1.202)	1.099 (0.997,1.212)
	Middle school and above	1.185*** (1.104,1.272)	0.973 (0.863,1.098)
Number of children		0.973** (0.953,0.994)	0.996 (0.962,1.032)
Child Education (Primary or below)	Junior high School	1.005 (0.919,1.100)	1.125 (0.969,1.306)
	Senior high school and above	1.213*** (1.107,1.330)	1.166** (1.001,1.359)
Child Residence (Not living with any child)	Living with at least one child	0.959 (0.914,1.005)	0.905** (0.837,0.978)
Marital status (Unmarried)	Married	0.820*** (0.747,0.900)	0.978 (0.840,1.138)
Insurance (Urban employee medical insurance)	Urban and rural resident medical insurance	0.515*** (0.459,0.578)	0.673*** (0.555,0.817)
	Others	0.556*** (0.410,0.771)	0.722 (0.380,1.372)
	No insurance	0.229*** (0.197,0.266)	0.312*** (0.241,0.404)
ADL (No difficulty)	Difficulty in at least one ADL item	1.193*** (1.113,1.278)	1.317*** (1.175,1.476)
Drinking behavior (No drinking)	Non-continuous drinking	1.211*** (1.145,1.281)	1.208*** (1.097,1.329)
	Continuous drinking	0.986 (0.920,1.057)	1.023 (0.910,1.150)
Smoking behavior (No smoking)	Non-continuous smoking	0.810*** (0.757,0.867)	0.864** (0.773,0.966)
	Continuous smoking	0.556*** (0.513,0.602)	0.556*** (0.487,0.636)

*P < 0.05, **P < 0.01, and ***P < 0.001, 95%CI= 95% confidence interval for the odds ratio.

Among sociodemographic factors, urban residents (OR = 1.241) and those with middle school education or above (OR = 1.185) were more likely to participate in continuous health examinations.

In contrast, married individuals showed lower participation (OR = 0.820, 95% CI: 0.747–0.900). The individuals with Urban and Rural Resident Insurance (OR = 0.515), other types of insurance (OR = 0.556), and no insurance (OR = 0.229) showed a significantly lower likelihood of participation than that of those covered by urban employee medical insurance. Family-level factors and functional status were also associated with participation. An increased number of children was associated with a lower likelihood of continuous participation (OR = 0.973, 95% CI: 0.953–0.994), while having children with a senior high school education or above was associated with higher odds (OR = 1.213, 95% CI: 1.107–1.330).

Building on this, Model 7 applied a localized RDD focusing on individuals aged 60–70 years and showed that although participation likelihood was higher after age 65 (OR = 4.223), the association was not statistically significant (95% CI: 0.156–114.389; *P* = 0.526), suggesting a limited effect of the current policy’s age threshold. Notably, ADL limitations (OR = 1.317), urban residence (OR = 1.400), high child education (OR = 1.166) and co-residence with children (OR = 0.905) remained significantly associated with continuous participation.

Continuous health examinations were significantly associated with a lower likelihood of high-burden CCD trajectories, after adjusting for key demographic variables among older adults with a low baseline burden. Specifically, individuals with continuous health examinations were less likely to be in the Health Risk group (OR = 0.456, 95% CI: 0.278,0.748, *P* < 0.01). However, the associations for the Moderate Chronic Disease Growth and Multiple Chronic Diseases Worsening groups were not statistically significant. The detailed regression results are presented in Table 5.

**Table 5:** Associations between Continuous Health Examinations and CCD Trajectories in Older Adults Aged 65 and Above Adjusted for Baseline Chronic Disease Status.

Variables	Options	Chronic Disease Latent Class		
		Model 8	Model 9	Model 10
		OR (95% CI)		
		Health Risk Group (Reference: Health Maintenance Group)	Moderate Chronic Disease Growth Group (Reference: Moderate Chronic Disease Slow Growth Group)	Multiple Chronic Diseases Worsening Group (Reference: Multiple Chronic Disease Retention Group)
Examination (Participated in health examinations but not continuously)	Participated in health examinations continuously	0.456** (0.278,0.748)	0.824 (0.583,1.163)	1.312 (0.886,1.944)
Sex (Male)	Female	0.989 (0.545,1.794)	1.063 (0.716,1.578)	1.088 (0.687,1.722)
Age		0.990 (0.950,1.032)	0.983 (0.955,1.012)	0.983 (0.949,1.018)
Urbanity (Rural)	Urban	0.994 (0.493,2.004)	1.009 (0.602,1.692)	1.418 (0.810,2.482)
Education level (No formal education)	Primary school	1.169 (0.700,1.952)	1.464* (1.024,2.094)	1.016 (0.675,1.529)
	Middle school and above	1.048 (0.496,2.213)	1.819* (1.098,3.014)	1.207 (0.670,2.174)
Number of children		0.970 (0.839,1.123)	0.983 (0.887,1.090)	0.957 (0.851,1.077)
Child Education (Primary or below)	Junior high School	1.163 (0.671,2.016)	1.188 (0.804,1.755)	0.893 (0.569,1.402)
	Senior high school and above	0.966 (0.541,1.724)	1.188 (0.786,1.795)	1.122 (0.690,1.822)
Child Residence (Not living with any child)	Living with at least one child	0.867 (0.572,1.314)	1.256 (0.941,1.677)	0.867 (0.617,1.219)
Marital status (Unmarried)	Married	0.997 (0.603,1.648)	0.920 (0.652,1.299)	1.289 (0.873,1.905)
Insurance (Urban employee medical insurance)	Urban and rural resident medical insurance	0.785 (0.332,1.856)	1.308 (0.724,2.365)	1.297 (0.678,2.483)
	Others	-	-	-
	No insurance	0.309 (0.081,1.182)	0.930 (0.255,3.391)	0.611 (0.176,2.123)
ADL (No difficulty)	Difficulty in at least one ADL item	2.114* (1.148,3.895)	1.312 (0.925,1.860)	1.200 (0.840,1.714)
Drinking behavior (No drinking)	Non-continuous drinking	0.898 (0.621,1.779)	0.895 (0.639,1.254)	0.962 (0.654,1.415)
	Continuous drinking	0.396** (0.220,0.715)	0.726 (0.473,1.114)	0.519* (0.290,0.928)
Smoking behavior (No smoking)	Non-continuous smoking	1.051 (0.621,1.779)	0.691 (0.477,1.001)	1.066 (0.685,1.659)
	Continuous smoking	0.685 (0.317,1.480)	0.862 (0.476,1.560)	0.887 (0.420,1.874)

*P < 0.05, **P < 0.01, and ***P < 0.001, 95% CI= 95% confidence interval for the odds ratio.

A sensitivity analysis was conducted by assigning higher weights to patients with cancer, kidney disease, and stroke based on the logic of the Charlson Comorbidity Index (CCI), while also incorporating age weights, to ensure robustness. The results showed that the weighted index was highly correlated with the original count (*r* = 0.89, *P* < 0.001), and both LCGA and RDD findings remained consistent.

## Discussion

According to the analysis, continuous health examination behaviors showed a complex relationship with CCD trajectories. Rather than exerting a universally protective effect, continuous participation was positively associated with higher-burden CCD trajectories in unadjusted models. Continuous health examinations showed a stronger association with stable health trajectories for those with low or moderate baseline chronic disease burden among individuals aged 65 and above. China’s free health examination policy has played an important role in encouraging participation and improving CCD management, but the association of health examinations was weaker among individuals with higher baseline disease burden. These results imply that the effectiveness of continuous health examinations is highly heterogeneous, profoundly moderated by baseline health status, policy frameworks, and individual socio-behavioral factors.

Our primary finding reflects that continuous health examinations do not directly prevent CCD progression, and this aligns with ongoing global debates regarding the efficacy of general health screenings. Although health examinations are valuable for detecting health problems, their main role is early identification rather than the prevention or treatment of existing conditions. Lasse et al.^20^ found that routine examinations in western populations do not significantly reduce disease incidence. We also found that the association between continuous health examinations was more pronounced among low-risk groups, contrasting with findings by Fragala et al.,^21^ which emphasized the preventive role of early detection in high-income countries. The difference may reflect increasing chronic disease burden over time,^22^, ^23^ compounded by a critical global health divergence in preventive care. In many high-income countries, lifelong routine screenings are deeply integrated into primary care. In contrast, in developing nations like China, older adults often lack a lifelong history of regular preventive care. Our observation of a positive association between continuous health examinations and unfavorable disease trajectories may reflect these patterns, where health examinations improve early detection, especially in asymptomatic cases, which could create a “detection effect”.^24^ Furthermore, low health awareness and limited proactive exam behavior^25^ in China may lead individuals with poorer health to participate in continuous health examinations, possibly resulting in reverse causality.

Our analysis reveals that the relationship between continuous health examinations and CCD trajectories is highly stratified by baseline chronic disease status, extending beyond the initial detection effect. After controlling for baseline CCD levels, both non-continuous and continuous health examination participants showed a higher likelihood of being classified into higher-burden CCD trajectories than that of those who never participated, possibly due to a lack of awareness of their chronic conditions.^22^ After adjusting for age and excluding individuals who had never participated in health examinations, continuous health examinations were still significantly associated with a lower likelihood of unfavorable CCD trajectories among individuals with a lower baseline CCD burden. Contrastingly, continuous health examinations did not show a significant association with stable trajectories for individuals with a moderate baseline CCD burden.^15^, ^26^ This may be because this subgroup is already more health-conscious and regularly engage in health management behaviors.^27^ Additionally, continuous health examinations did not show a significant association with reduced CCD burden, likely due to the severity of their pre-existing health conditions among individuals with high baseline CCD burden.

At the macro-policy level, China’s free health examination policy has effectively promoted continuous examination participation, supporting CCD management. RDD analysis showed that individuals aged 65 and above were significantly more likely to undergo continuous examinations, indicating a positive role of the policy. Continuous participation was associated with a lower likelihood of belonging to higher burden CCD trajectories among those with a low or moderate baseline CCD burden. However, the potential benefits of the policy are constrained by its late eligibility age: many people already have multiple chronic diseases by 65. Moreover, a localized RDD analysis among individuals aged 60–70 further found no significant increase in examination rates at age 65. The wide confidence interval suggests that the immediate impact of the policy at this threshold is statistically inconclusive in our localized analysis, possibly due to delayed policy exposure at baseline.^28^ These results may imply that the 65-year threshold is not the optimal point of intervention, as restricting eligibility may not fully capture the preventive potential of the program. The inclusion of younger age groups could foster early detection, promote long-term examination habits, and improve CCD management. Moreover, the optimization of this age threshold offers a valuable blueprint for other low- and middle-income countries seeking to maximize the cost-effectiveness of preventive healthcare resources before populations age into high-burden morbidity.

The outcomes associated with China’s free health examination policy are profoundly shaped by socio-demographic disparities, health literacy, and system-level factors, extending beyond age thresholds. While urban residents and those with higher education were more likely to engage in continuous health examinations, urban participants with high baseline CCD burden were more likely to be classified into higher-burden CCD trajectories—possibly due to lifestyle-related factors.^27^, ^29^ The individuals covered under urban employee medical insurance were more likely to engage in continuous health examinations.^30^ In addition, some individuals have difficulty understanding their examination results, which may limit the integration of health information into their daily behaviors.^31^ Low health awareness and inadequate follow-up interventions may limit the potential benefits associated with examinations.^32^, ^33^ This underscores that without parallel improvements in health literacy, merely eliminating financial barriers severely limits the preventive value of free screenings, aligning with Universal Health Coverage (UHC) goals in developing nations. Therefore, efforts to increase health awareness and health literacy are crucial to promoting continuous health examination participation and fostering a positive cycle of health-promoting behaviors.

Furthermore, the negative association between alcohol and high-burden CCD trajectories may reflect survivor bias and the sick quitter effect,^34^ where individuals with poorer health cease drinking, rather than a true protective benefit.

Some limitations also need to be considered. The use of self-reported data may introduce measurement and detection biases, potentially leading to recall bias and data inaccuracies; however, previous research does support the validity of self-reported chronic disease diagnoses.^35^ Furthermore, the analysis focused on health examination frequency, without accounting for subsequent care, lifestyle changes, or treatment adherence, which may influence CCD outcomes. Additionally, the latent class analysis relied solely on disease counts, omitting disease type, duration, and severity. The 2020 wave also coincided with the COVID−19 pandemic, which may have disrupted preventive health services and health behaviors, potentially influencing health examination participation and chronic disease identification. However, the sensitivity analysis excluding 2020 data showed consistent trajectory patterns. Lastly, selection bias and reverse causality should be considered. Although the CHARLS database uses PPS sampling to reduce bias, selection bias remains possible because our study focused on middle-aged and older adults.^36^ As individuals with poorer baseline health may be more likely to undergo continuous examinations, this self-selection process could contribute to the observed associations in certain trajectory groups. Longitudinal studies are needed in the future to prove causal associations.

## Conclusions

This study demonstrates that the impact of continuous health examinations on CCD trajectories is highly contingent upon baseline health status. Notably, relying on a 65-year threshold for free screenings is suboptimal; lowering the eligibility age is critical to intercepting disease progression early. Healthcare systems should adopt risk-stratified interventions by prioritizing screenings for low-risk groups and multidisciplinary care for high-burden individuals. Health literacy strengthening and proactive primary care follow-ups are also essential to translate screening results into sustained behavioral change. Together, these targeted strategies can guide developing nations striving to optimize preventive healthcare resources amid rapid population aging.

## List of abbreviations

CCD: Comorbidity of chronic diseases

CHARLS: China Health and Retirement Longitudinal Study

## CRediT authorship contribution statement

**Dan Han:** Writing – review & editing, Methodology. **Sangsang Li:** Writing – review & editing, Visualization. **Shiping Gu:** Writing – review & editing, Project administration, Resources. **Xinnan Du:** Writing – review & editing, Methodology, Visualization. **Yixuan Zhong:** Writing – review & editing, Visualization. **Yizhou Ao:** Writing – original draft, Formal analysis, Data curation, Conceptualization, Methodology, Visualization. **Jing Wang:** Writing – review & editing, Supervision, Methodology, Funding acquisition, Conceptualization.

## Declaration of Generative AI and AI-assisted technologies in the writing process

Statement: During the preparation of this work the authors used ChatGPT in order to improve the readability and language of the manuscript. After using this tool, the authors reviewed and edited the content as needed and take full responsibility for the content of the published article.

## Consent for publication

Not applicable.

## Ethics consideration

All methods used in this study were implemented in accordance with relevant CHARLS guidelines and regulations. All participants joined CHARLS voluntarily and signed a consent form before participation. The original CHARLS was approved by the Ethical Review Committee at Peking University in June 2008 (IRB00001052–11015).

## Funding

This work was supported by the 10.13039/501100001809National Natural Science Foundation of China (grant numbers 72574074); the 10.13039/501100001809National Natural Science Foundation of China (grant numbers 72074086).

## Declaration of Competing Interest

The authors declare that they have no known competing financial interests or personal relationships that could have appeared to influence the work reported in this paper.

## Data Availability

The data used in this study are publicly available from the China Health and Retirement Longitudinal Study (CHARLS) database (http://charls.pku.edu.cn/)

## Footnotes

Appendix A Supplementary data associated with this article can be found in the online version at doi:10.1016/j.ghrp.2026.06.006.
