# Supplementary material for: Associations between continuous health examinations and trajectories of comorbidity of chronic disease: Evidence from a nationwide longitudinal study of middle-aged and older adults in China
Source: Glob Health Res Policy. 2026 Jun 29;11(1):120–30. doi: 10.1016/j.ghrp.2026.06.006 (PMC13544087; doi:10.1016/j.ghrp.2026.06.006)
Supplement: Supplementary file 1 [file mmc1.docx]

**Supplementary Table 1 Coding of variables**

| **Variable** | **Coding** |
| --- | --- |
| Comorbidity of chronic diseases Latent Class | Trends in the number of chronic diseases among respondents from 2013 to 2020  Health Maintenance Group =1  Health Risk Group =2  Moderate Chronic Disease Slow Growth Group =3  Moderate Chronic Disease Growth Group =4  Multiple Chronic Disease Retention Group =5  Multiple Chronic Diseases Worsening Group =6 |
| Examination | Never participated in health examinations = 0  Participated in health examinations but not continuously = 1  Participated in health examinations continuously = 2 |
| Sex | Female=0, Male=1 |
| Age | Continuous variable |
| Urbanity | Rural=0, Urban=1 |
| Education level | Illiteracy = 0, Primary school= 1, Middle school and above = 2 |
| Number of children | Continuous variable |
| Child Education | Primary or below = 0, Junior high School = 1, Senior high school and above = 2 |
| Child Reside | Not living with any child=0, Living with at least one child=1 |
| Marital status | Unmarried=0, married=1 |
| Insurance situation | Urban employee medical insurance=1  Urban and rural resident medical insurance=2  Others=3  No insurance=4 |
| ADL | No difficulty=0, Difficulty in at least one ADL item=1 |
| Drinking behavior | No drinking=0  Non-continuous drinking=1  Continuous drinking=2 |
| Smoking behavior | No smoking=0  Non-continuous smoking=1  Continuous smoking=2 |

**Supplementary Table 2 Distribution of Baseline Chronic Disease and Changes in Comorbidity of chronic diseases**

| Baseline chronic diseases | Change in Comorbidity of chronic diseases | | | |
| --- | --- | --- | --- | --- |
|  | 0 | 1-2 | >3 | Total |
| 0 | 1,332  (29.7%) | 2,083  (46.52%) | 1,063  (23.74%) | 4,478 |
| 1-2 | 2,129  (35.44%) | 2,563  (42.67%) | 1,314  (21.88%) | 6,006 |
| >3 | 811  (40.03%) | 780  (38.50%) | 435  (21.47%) | 2,026 |
| Total | 4,272 | 5,426 | 2,812 | 12,510 |

**Supplementary Table 3 Results of Latent class** **growth analysis**

| **Model** | **AIC** | **BIC** | **Adjusted BIC** | **VLMR-LRT**  **(p)** | **BLRT(p)** | **Entropy** | **Sample Proportion(%) per Class** |
| --- | --- | --- | --- | --- | --- | --- | --- |
| 1 Class | 177851.994 | 178119.628 | 178005.224 |  |  |  |  |
| 2 Classes | 157370.562 | 157913.265 | 157681.278 | 0.000 | 0.000 | 0.921 | 41.71/58.29 |
| 3 Classes | 148005.697 | 148823.468 | 148473.900 | 0.000 | 0.000 | 0.915 | 28.65/41.04/30.31 |
| 4 Classes | 142691.087 | 143783926 | 143316.776 | 0.010 | 0.000 | 0.962 | 21.91/28.67/30.08/19.34 |
| 5 Classes | 139921.693 | 141289.601 | 140704.868 | 0.000 | 0.000 | 0.945 | 12.10/12.22/29.65/18.43/27.60 |
| 6 Classes | 137195.294 | 138838.271 | 138135.956 | 0.000 | 0.000 | 0.937 | 23.73/13.65/11.97/11.90/20.44/18.31 |
| 7 Classes | 135004.059 | 136922.104 | 136102.207 | 0.681 | 0.000 | 0.937 | 18.32/13.55/16.55/11.98/13.96/14.24/11.41 |

*AIC: Akaike information criterion, BIC: Bayesian information criterion, LMR: Vuong-Lo-Mendell-Rubin, BLRT: Bootstrap-based likelihood ratio test index.
